# Supplementary material for: Chromothripsis during telomere crisis is independent of NHEJ, and consistent with a replicative origin
Source: Genome Res. 2019 May;29(5):737–49. doi: 10.1101/gr.240705.118 (PMC6499312; doi:10.1101/gr.240705.118)
Supplement: Supplemental Material [file supp_gr.240705.118_Supplemental_file_1.zip › contigs/annotated_contigs/DB45/contig.3.DB45_length_1247_mean_cov_10.1491579791.docx]

**DB45_length_1247_mean_cov_10.1491579791**

```
CAATTTTGCAAATTTTCTCAGCACAAATGAAACATTTGATCACATTGCTAAGGCCCTTCCAAGGCCCTGGAATGTGTACTTGCCAACAA
 >chr8:138844219-138844627 - E=3e-233
GATGAGTTAGAGTTTAAGTTTTATTATCTTTCCAATAGTTTGCCGGGTCCAGTTCAAAATAAAAATGTGAGGCCCTTTGTTCTTAAAGC

ATTAAAATTATCAAGGAATACATCCAGACTGTGAGCATCAGAAAAAACACCTGAAGACATTGAGCCTATGTCCATGGGTAATGACAGAT

GAGCATGACAGGAGCTTCAGAGTGAATAGATCCTTATCCTCTTTAGCAGCTCTCAATTGCAAATTTTCTAGGGAAGATCCCAGGAAGGT

ACCTGCTGGCTTTAAAGCAAACTCTTCTACAGGGATGGGAAAGCATGTAGC|A|GACCTTGATAAAATTACTTATAACATCAAAGCGTG
                                                    >chr8:139040330-139040758 - E=3e-24
GGCTTTATTTGCTTTTTCTCACCCAAGCGTGAGAATCCCTCTTACTCATGCACACATTTATGCTGCTGTTCTCTCCTTCCGCATCTCTT
5 p=1e-03
ACAGGACAAAGAATGGGTTTTCTTTTCACATTCCTTCTTATTTCTAAGGGGGAAAAATGTATTTCTTTCTCACCTCATAGTGTGAGCTC

CAAATCAGTTATGATGATCGGCCCAACCCTTGAGCATCTGTGCTTAGTGACACAACGCCCATTGTACTAGCCTTGCACCCCTTCTGCCT

TGCTTGCTGACATCCTAATAAAACCACTCAATTCTCTTTTGTTCATCCCATGCACTGTCCCACAGTGAACTCCATCCTTGGTGTTTTGC

AACGGTCCACAGAAATGGAATAAAACTGTTGATA|TC|GAGAATTCCTGCCCTACTTGATGATGCCTCCGTATTGCAGTTAGCAAAACT
                                   >chr8:139040846-139041201 + E=2e-198
ATACATGAATTCCACAAGGAGTCTATGGATTGTCATTAAGACATCAGAAATATTTTAATTGTATTCAAAATTATGTGTTTGTGTGAATG

CACACATTTTGATAGAGGAGAGAAGTTTGCAGAGATTTCATAAATTTCTTGAAGGAGACTAAGTTCCCAAATATTGAAGAAACACTGTC

AGACAGTATTGATTTTTCCTACGTTCCCCTATGTACCTGATTTGGGTTTTCATCACATAGCTCAGGTATCTTTGAAACTGACATTTCTT

TCTTTTCTTTTTCTTTCTTTTTTTTTTTTTTTTTT|GTTGGTGTTTTGTTTTGTTGTCCAGGCTTGTGTGCAATTGCTGGTTCTTAGCT

CACTTG
```
